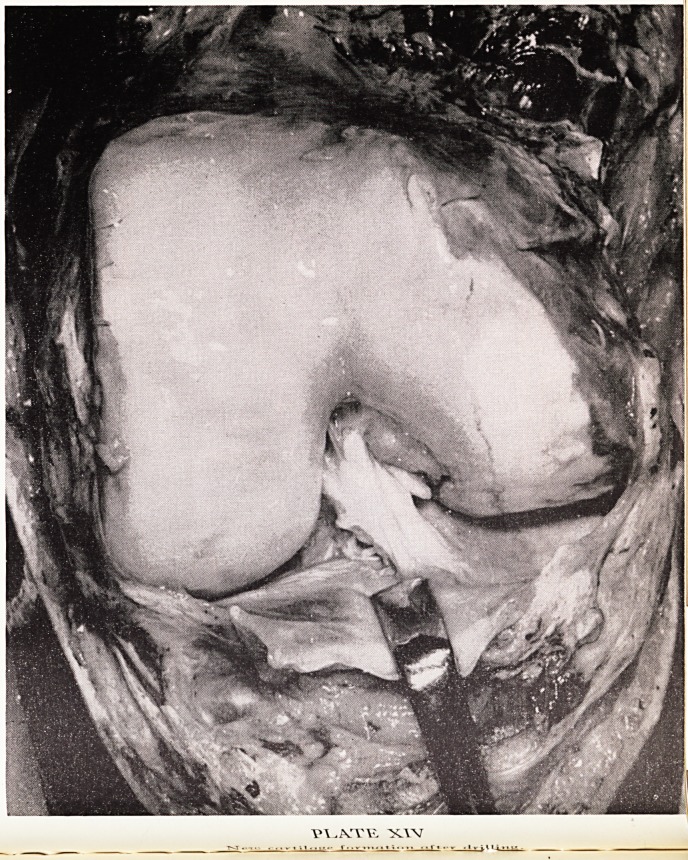# Kenneth Pridie: An Appreciation
*The first Kenneth Pridie Memorial Lecture, delivered at Bristol on 8th April 1965 before members of the University of Bristol and of the British Orthopaedic Association.


**Published:** 1965-07

**Authors:** A. L. Eyre-Brook


### KENNETH PRIDIE: AN APPRECIATION*

BY

A. L. EYRE-BROOK

This is the first Kenneth Pridie Memorial Lecture and I deem it a great honour to have been invited to give it. The honour might well have fallen to someone really eminent in the world of orthopaedic surgery, but this inaugural address needs to be personal to the surgeon and to the man whom we honour this evening; and as his closest colleague for 25 years I felt I must accept this invitation. It is particularly pleasing to me and to the many who have striven to produce this memorial that the first lecture should take place before the British Orthopaedic Association on their third visit to Bristol, so that many of the Fellows and Members who have assisted in this worthy venture should be present, more especially as I intend to devote my remarks entirely to the work and to the personality of Kenneth Pridie. Let me attempt to portray the latter first.

Properly to convey the personality it is incumbent on me to strike a note of cheerfulness, courage, vigour and inventiveness. Nothing was further from Kenneth Pridie and his life and his surgery than austerity, pomp or smallness. He was the younger son of a doctor, born into a family of four and he lost his father at the age of 23, just after he himself qualified in 1928. His sister writes that they had a very strict upbringing but that Kenneth could get away with anything and it was always refreshing in times of tension to have someone to laugh at. At a certain meeting at the Royal Society of Medicine just after the war, when Mr. Pridie demonstrated three cases of excision of os calcis, he turned to me and said with a laugh "They always put me on as the funny turn".

At University Mr. Pridie already excelled in the field of sport, being the University heavyweight boxing champion and a fine performer on the rugby field as well as in athletics, but his greater accomplishments came later in shot-putting and with the discus, in which he represented England and broke the native record.

His early surgical training was characteristically unorthodox—a number of three-months appointments, a trip as ship's surgeon on a multi-millionaire's yacht, and two six-months appointments in surgery, were followed by visits to Dr. Bohler's Clinic in Vienna, to Sir Reginald Watson Jones's Fracture Clinic in Liverpool, and by a short stay with Mr. Girdlestone in Oxford. He returned to Bristol and obtained the appointment of Assistant Fracture Surgeon at the Bristol Royal Infirmary soon afterwards, in 1936.

His closest association with Hey Groves occurred during the next few years and I am certain that he would have wished me to indicate how much he owed to Mr. Hey Groves who, however, had retired before Mr. Pridie's return to Bristol. Mr. Hey Groves's appreciation of his young colleague in a testimonial concludes with the sentence . . . "His character and personality are such as to contribute life and vigour to any Institution to which he is attached". Mr. Chitty at this same period sums up Mr. Pridie . . . . "His originality of mind, his enthusiasm and his practical ability make it a pleasure to work with him", and both these opinions were amply justified during the subsequent 30 years.

Mr. Pridie married in 1934 and no remarks on the life and works of this man can proceed far without referring to his charming wife Joanna, whose other qualities are

* The first Kenneth Pridie Memorial Lecture, delivered at Bristol on 8th April 1965 before members of the University of Bristol and of the British Orthopaedic Association.

evident from the happiness of their home and family life. The house in St. John's Road was such a friendly yet lively home, sometimes almost over-full of children, but always buzzing with ideas and plans—boat-building, holiday plans, or schemes for the two family playgrounds, the Chalet and the Scilly Isles, both providing plenty of opportunity for the vigorous outdoor pursuits that characterized the Pridie way of life. At the Chalet tree-felling, hut-building, tractors, and a saw-mill vied with each other to claim the interest of visiting friends during daylight hours, and some will have enjoyed the evening barbecues, with Mr. Pridie in charge of the cooking.

Ken Pridie's interests were legion; from rugby to chess, from books on the structure of the atom to three months in Iceland with the schoolboys; he was no narrow modern specialist. At all times he was a stimulating companion. His infectious enthusiasm made one feel better for being in his company. He was never ashamed to ask questions or to confess ignorance, and in spite of a formidable exterior he always retained an engaging humility. He was better in opposition (where he instinctively found himself) than on the side of the Establishment; better as light relief than as one of the major speakers at a dinner, although in later years he was less unpredictable in this latter role, and would always give full value in humour of the very best type.

He was a forthright and colourful speaker, with a great aptitude for quotation, and a pleasant wit. In his early thirties he would often enliven the meetings of the British Orthopaedic Association. The sight of his massive form advancing towards the rostrum would stimulate flagging interest, and a smattering of over-statement would serve to whet the appetite. In his younger days he seemed to like the role of *enfant terrible*, and he would say "I bear with fortitude the sufferings of my patients". He could give very sound advice; we could both write a strong letter full of righteous indignation, but on his advice I always put mine in the drawer, and only sent it if it read equally well in the morning. It rarely did. Ken Pridie used to tell me that "It would be a bad day when we did not have too much to do", and he would add with a twinkle "We might start doing unnecessary operations".

It is my main purpose here to take stock of Pridie's contribution to orthopaedic surgery, and I shall try to follow his interests and innovations in chronological order over thirty years. In his early days he enthusiastically espoused Böhler's technique in the treatment of fractures, and he tried to get his patients out of hospital with the fractured bone transfixed with a Kirschner wire and stirrup. Kirschner stirrups were sometimes incorporated in the plaster and lost sight of, and I once saw the gleaming metallic section of a stirrup he had just cut through while removing the plaster. It was one of those occasions when he seemed particularly warm from his exertions.

Pridie was always a great advocate of the Hamilton Russell traction, and he devised an excellent beam to replace the heavy Balkan beam then in use. The ease with which his beam could be transported and used won many admirers. The grapple irons fitted as easily to the bars of a hospital bed as to the solid end of a bed in a cottage where a patient with a pathological fracture of the femur could be made comfortable for her remaining months of life. Square fitments, to obviate too much reliance on the thumb-screw, were an ingenious mechanical introduction; the slides ran easily along these bars but did not rotate.

During the War years he became very interested in cup arthroplasty. The presence of American hospitals in Britain during the War gave him a good deal of contact with American orthopaedic surgeons. Shortly after the War, Mr. Pridie designed an excellent ball-cutter for hip arthroplasty with the aid of the then Bristol Aeroplane Company. As was typical of his inventions, the instrument was simple and robust in design. It is certainly, in the opinion of many, the best instrument in this field. After a brief period in which, like many orthopaedic surgeons, he was infatuated with the slender acrylic prothesis from Paris, he returned to his cups, finding this a far more manly and reliable pursuit when faced with a hip arthroplasty. The arresting title of

PLATE XII

*Kenneth Pridie at Winford Hospital.*

[*facing p.* 38

PLATE XIII

Multiple drilling of eburnated femoral condyle.

PLATE XIV

New cartilage formation after drilling.

his paper in the Postgraduate Journal immediately after the War, "New Hips for Old", was a forceful inducement to submit to an arthroplasty.

Being essentially an innovator and experimenter, he was forever probing fresh methods for hip arthrodesis; and even in his later years he would approach the new venture with the enthusiasm of youth. Life was never dull for his colleagues or his assistants; change was ever with us, although the assessment of the present might be inadequate as he moved forwards into the rosy prospects of the future.

During the War years Mr. Pridie advocated the excision of the os calcis in very severely comminuted fractures, those destined to cause immense swelling. The idea was not put forward as a panacea in all these difficult fractures, indeed the operation was reserved for the worst group, and throughout subsequent years such an excision has occasionally been done; but most cases are still dealt with by a completely conservative mobilizing programme.

I must now refer to other instruments that Mr. Pridie used, adapted, or invented. Some of them were formidable and matched his physique, and his courage was equally in evidence; but he was an extraordinarily safe surgeon and very rarely had a disaster.

The Forstner augur bit was a carpenter's instrument which Mr. Pridie converted to surgical use. Ideal for his fusion of the ankle, it is a valuable tool in central dislocations of the hip; with the central spike cut back markedly, it is a delicate cutting instrument which I prefer to all others when fusing the cervical spine from the front.

In Pridie's fusion of the ankle, the 1 in. Forstner augur bit was used to drill transversely across the ankle from the inner side. Centred on the highest point of the curve of the talus, it would remove bone and cartilage as it travelled across to enter the fibula on the lateral side. The cylindrical cavity was then filled with new cancellous bone slithers removed with the same instrument from the lower third of the tibia, and added to the good bone removed from the internal malleolus at the original drilling. Very effective and rapid fusion of the ankle results from this cancellous packing, without major disturbance or mobilization of the arthritic joint. Many have found this a valuable operation to add to their repertoire.

The fine Forstner augur bit was used by Mr. Pridie in anterior fusions of the cervical spine, and is a delicate instrument with which to cut away the disc remnants and the contiguous bone of the vertebrae. A cutter removes a dowel of cancellous bone from the ilium. This dowel is a slightly large fit for the hole cut by the augur bit in the cervical spine, and it is forced into place after some distraction of the cervical spine by pulling on the head with a halter. A very tight fit and good fusion result.

The staple was another device that Mr. Pridie enjoyed using in fusion of the knee, as well as in retaining reduction of dislocations of the cervical spine.

Mr. Pridie characteristically espoused the Kuntscher nail for the femur, and later was encouraged in its widespread use in fractures of the tibia by Mr. Alms, who visited Dr. Kuntscher's Clinic on a Robert Jones Travelling scholarship. Mr. Alms's work has received due recognition, but he was constantly encouraged by Mr. Pridie. The use of the nail was extended to low fractures of the femur, and Mr. Pridie introduced it through the knee, a departure I could never favour because it can drop back. All surgeons have met their difficulties with the Kuntscher nail. People sometimes say with reason that if one should become a patient it is important to choose a lucky surgeon—and Ken Pridie also had this quality. I cannot leave the subject of Kuntscher nails without mentioning the effective extractor designed by Mr. Pridie for removing an obstinate nail from the femur. With this he used a 4-pound hammer, which could play a persuasive part, particularly with Mr. Pridie behind it. It behoves me to say at this point that he was a gentle operator, and I think the restrained use of a heavy hammer is much more controlled than the more violent use of a light one.

I now come to what is probably the most valuable of Mr. Pridie's contributions to orthopaedic surgery—his more recent work on the knee.

First, a passing reference to his treatment of the larger lesions of osteochondritis dissecans, where the replacement technique advocated by Mr. Smillie is not applicable because of the size and fragmentation of the lesion. Mr. Pridie would remove the sclerotic wall of the lesion down to healthy bleeding bone, fill the cavity with cancellous bone removed from the upper end of the tibia, and cover the surface with a layer of cartilage shaved off the femoral condyle, or perhaps partly from the discarded fragments. Then would follow six weeks immobilization of the knee, in extension, during which time the tibial articular surface would provide effective pressure on the cartilage graft overlying the cancellous bony filling. Movement would then be commenced and full function would soon return. To his constant chagrin it never happened that re-exploration was warranted; a very satisfactory outcome, though frustrating to the experimental surgeon not willing to indulge in unnecessary operations.

Shortly after the War Mr. Pridie became interested in that Cinderella, osteoarthritis of the knee. It was Magnussen's work that interested him, and he started with a restricted operation of partial synovectomy and chielotomy, shaving off the fibrillated articular cartilage; sometimes patellectomy was added. With maturing experience he had developed a very successful procedure by the time of his premature death. He found that the exposure advocated by Coonse and Adams in 1943 was of great help, giving a very extensive field of operation. Synovectomy was done less regularly, and more attention was given to the femoral condyles with their eburnated surfaces and dense underlying bone. These surfaces were drilled so as to bring vascular tissue to the ischaemic sclerotic surfaces. Later, the drilling was extended to the tibial plateaux.

Many successes were naturally accompanied by some cases with less gratifying results, and a fusion was sometimes called for, so that in a small minority Mr. Pridie was able to see the appearance of the knees months or years after the operation. Even in these, the changes were surprising, and accounted for the many satisfied patients in whom he had no opportunity to inspect the treated femoral condyles.

The beautiful photographic records collected by Mr. Pridie enable us to follow the stages of the operation in a typical case. On exposure of the joint, osteophytic ridging, fibrillated cartilage, loss of cartilage and eburnation of a femoral condyle would be evident. The osteophytic ridging was removed and the affected cartilage shaved away; where cartilage had been worn away the femoral condyle was drilled in several places. This drilling through the sclerotic subchondral bone reaches the vascular cancellous bone, and was designed to allow vascular tissue to come to the surface. When, at this point in the operation, the tourniquet was removed, bleeding was seen from the fenestrations. Immobilziation for 10 days was followed by mobilizing physiotherapy, and probably a manipulation would be needed 4 to 6 weeks after the operation, to assist in getting movements back in the knee. Rehabilitation would need to continue for 3 or 4 months, but would yield a result satisfying to the surgeon and the patient in its painlessness and adequate range of movements.

A knee which failed to give a good result, and therefore allowed of inspection at a subsequent operation, was very instructive. In one case there were originally severe changes, gross eburnation of the condyles and a meniscus lesion. At the end of the first operation the femoral condyle somewhat resembled a sieve; but two years later, when the knee was re-explored, the fibro-cartilagenous circles over the perforations showed up as some of the most normal and smooth portions of the femoral condyle, and they were fusing to form confluent sheets. In another very instructive case, the knee showed very severe osteoarthritic changes with "tramlines" localized to one condyle. All the osteophytic ridging was removed, fibrillated cartilage was shaved, and drilling was performed through all the eburnated bone (Plate XIII). Four months later the patient continued to complain and would not return to work, and so the knee was re-explored. It was now seen that a smooth white surface of fibro-cartilage had replaced the "tramlines" of eburnated bone (Plate XIV). Mr. Pridie proceeded to fuse the knee as the man had expressed a preference for a strong stiff knee rather than for the discomfort of his mobile knee, in view of his heavy occupation. Sections from the condyle showed the blending of the fibro-cartilage into smooth sheets, and it was only the radiographs of the section that showed the drill-holes made at the operation four months previously.

In the patient just described, fusion was carried out using staples, as was Mr. Pridie's practice. The undoubted advantage of this method is that no external splintage is needed; the disadvantage, however, is that staples sometimes become a little prominent and require removal later. This method of arthrodesing the knee proved very successful in Mr. Pridie's hands and enabled him to get his patients out of hospital quickly, an aim he always considered of paramount importance.

This work on osteoarthritis of the knee is being continued, and since Mr. Pridie died I have had more personal experience of the gratifying results that can be obtained from his operation on suitably selected patients. Results are delayed for 4 to 6 months, but many a sufferer from an osteoarthritic knee is very grateful for the offer of such an operation to give relief from his chronic complaints, and is willing to devote this amount of time for the purpose, particularly in view of the good prognosis we can give.

This brief survey of the more important contributions that Mr. Pridie made to orthopaedic surgery is now concluded, and the man being greater than his work, I should like to finish by referring to some appreciation of Mr. Pridie made in letters I have received. Some have admired his patent enjoyment of life, others stress his colourful personality, and to another he was "That shock-headed figure with his provocative ideas". Philip Wiles wrote that "He lived his life as he enjoyed it—and orthopaedic surgery was only a part; that is the way it should be".

Yes, a unique personality and a man of many parts. This vital being has crossed the stage of life and left us all the richer for knowing him.

It remains only for me to thank the University of Bristol and the South-West Orthopaedic Club for asking me to deliver this Lecture in honour of an old friend and colleague who has claimed my admiration and affection undimmed through the 30 years of our close association.

## Figures and Tables

**PLATE XII f1:**
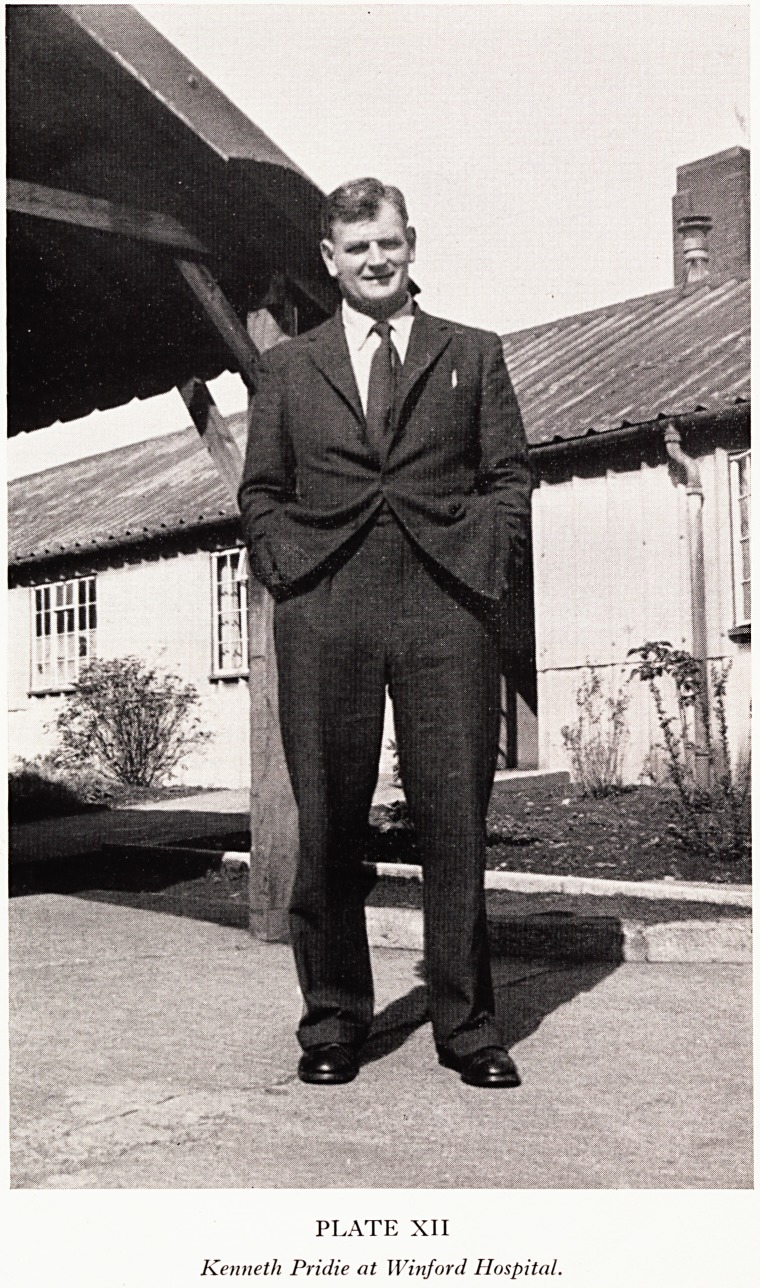


**PLATE XIII f2:**
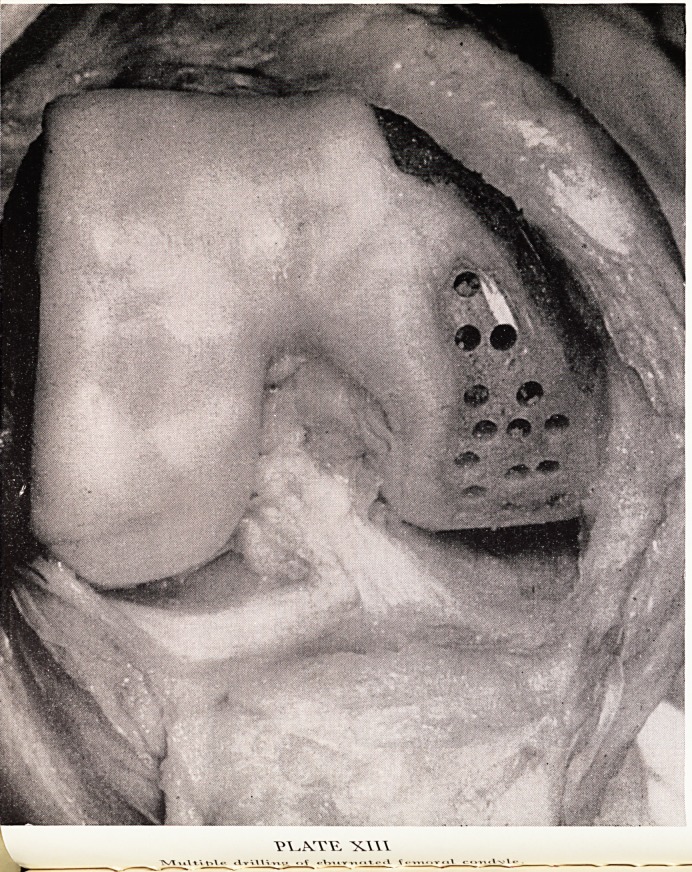


**PLATE XIV f3:**